# Annotations

**Published:** 1897-10-16

**Authors:** 


### Annotations.

### Sir Samuel Wilks.

A few of the old friends and pupils of Sir Samuel Wilks gave a dinner in his honour last week. We say a few—really they numbered more than have ever dined in the large hall at the Metropôle—but what is that among the multitude who, scattered in every quarter of the globe, are taken back by the name of Dr. Wilks to happy student days; to memories of his earnest and constant effort to separate fact from theory; the humour which he brought to play in demonstrating error; and, above all, the high tone of honour which he inculcated. When Sir Samuel Wilks, looking back from the post of honour and high dignity which he now occupies, says that he has never courted reputation or cared a straw for public opinion, and that the only thing he has cared for has been the respect of his medical brethren, we feel that he speaks only the simple truth. But we feel also that the world cannot be quite so rotten as some would have us think, when we see that, after all, the highest honours which his profession can offer, and high honours also from those who are great in the land, come in the end to such a man as Sir Samuel Wilks—a man who has not cared for them or worked for them, and, above all, has not asked for them, but has simply done his best in all the work that has come before him, and has done it in a way so full of sympathy with fellow-workers and charity to opponents, as to earn for himself the love and abiding respect of all with whom he has been brought in contact. We will not indulge in contrasts; but the world—and the medical world is no exception—is so full of push and self-seeking, of men whose thoughts, even in the midst of good work, run on place and influence and ambition, and who measure the honourableness of their profession by the honours which it brings, that it is a pleasure to turn to the thought of one who has bided his time and done his work, gathering to himself meanwhile the affectionate regard of many pupils—one whose public life has been devoted to duty and to science, and who now stands upon a pinnacle of honour.

### Blood-letting.

Are we going to see a revival of the use of bleeding as a means of treatment? It seems not unlikely. The fact must be confessed that medical men have for many years been far too greatly swayed by fashion. Dr. Carter, of Liverpool, only spoke the truth when he said, as he did a few days ago, that one of the great hindrances to progress in medicine arose from the dogmas uttered by its great men. Unfortunately the chief manufacturers of medical dogma have been the teachers practising in great towns, among people who undoubtedly did not bear depletion well, and among diseases of a septic and low type. Hence the fiat years ago went forth that bleeding was an abomination, a mere relic of the barbaric age; and as a result of this it may safely be asserted that there are hundreds, nay thousands, of men in practice who have never used a lancet for its primary purpose, namely, that of venesection. If the making of dogmas had been a country pursuit we may doubt whether the revulsion against bleeding would ever have become so complete as it has been for the last thirty or forty years. But the country has followed the town; bleeding has been looked upon as barbaric, and few practitioners have cared to ticket themselves barbarians. In a half-hearted way, no doubt, bleeding has been used in cases of suffocative broncho-pneumonia, and its use has never been entirely omitted in dealing with puerperal eclampsia. But it has been done with an apologetic air, and as something quite out of the common. There will probably, however, before long be a swing of the pendulum. Country practitioners are less ready to bow the knee; they have learned some science, they are trained observers, and by aid of the medical journals they keep themselves abreast of all that is going on. It is only to be expected, then, that they will argue out the treatment of their own cases for themselves, and that they will decline to treat lusty farmers on the same principles as were deduced from observations on the inhabitants of Clare Market. The arguments in favour of a small bleeding in the early stage of a sthenic lobar pneumonia are very strong, while in ingravescent apoplexy, occurring in great, strong, lusty countrymen, they are absolutely overwhelming. Yet how seldom do we see it done? We learn in great cities, and we carry city practice into country wilds. But this will not continue.

### Chilly Churches.

Self-denial is one of those virtues which we would say nothing against, and we would even admit that there are many people in the world to whom a little wholesome penance would do no harm. We feel much inclined, however, to doubt the wisdom of those ecclesiastical authorities who, by encouraging a wintry coldness in the house of God, make church-going a self-denying ordinance, and early services a form of penance. The chilliness of churches is, indeed, proverbial, and many are the colds which are caught by visitors to the sunny south while investigating the art treasures with which so many of these edifices are made attractive. To kill off a few ungodly globe trotters by pneumonia, the baccillus of which would appear to have a special predilection for Italian churches, may be a perfectly proper proceeding, especially after the verger has pocketed his *lira*, but that priests of the Anglican Church should deal thus with their fair but none too numerous devotees seems hardly worldly wise. At any rate it sets medical men against church-going, which is bad for both the doctors and the churches. The doctors, sadly be it said, have none too good a reputation in regard to forms and ceremonies, and when they in all honesty warn their patients against risks involved in certain religious exercises, they earn for themselves much undeserved obloquy, while undoubtedly the churches suffer both in congregation and in cash by the obvious truth of what the doctors say. Why the vergers, or the grave-diggers, or whatever officials are responsible for the firing-up, cannot be made to keep our churches warm is a mystery. It does not seem to dawn upon them how many hours it takes to make a building reasonably comfortable. They fire up with great energy when they once set about it, and by the time of evening service the place is stifling, but in the morning the devotions of the sick—nay, even of the healthy—are surrounded by many perils. Strange and pathetic stories could be told on the subject of "churchings" and early services, and even christenings are responsible for many little graves.

